# The Contribution of *Trichoderma viride* and Metallothioneins in Enhancing the Seed Quality of *Avena sativa* L. in Cd-Contaminated Soil

**DOI:** 10.3390/foods13152469

**Published:** 2024-08-05

**Authors:** Wiktoria Konieczna, Sena Turkan, Marzena Warchoł, Edyta Skrzypek, Grażyna B. Dąbrowska, Agnieszka Mierek-Adamska

**Affiliations:** 1 Department of Genetics, Faculty of Biological and Veterinary Sciences, Nicolaus Copernicus University in Toruń, Lwowska 1, 87-100 Toruń, Poland; wpyrkosz@doktorant.umk.pl (W.K.); senatrkn47@gmail.com (S.T.); browsk@umk.pl (G.B.D.); 2 Centre for Modern Interdisciplinary Technologies, Nicolaus Copernicus University in Toruń, Wileńska 4, 87-100 Toruń, Poland; 3 The Franciszek Górski Institute of Plant Physiology, Polish Academy of Sciences, Niezapominajek 21, 30-239 Kraków, Poland; m.warchol@ifr-pan.edu.pl (M.W.); e.skrzypek@ifr-pan.edu.pl (E.S.)

**Keywords:** cadmium, heavy metals, metallothioneins, oat, phytoremediation, *Trichoderma*

## Abstract

Pollution of arable land with heavy metals is a worldwide problem. Cadmium (Cd) is a toxic metal that poses a severe threat to humans’ and animals’ health and lives. Plants can easily absorb Cd from the soil, and plant-based food is the main means of exposure to this hazardous element for humans and animals. Phytoremediation is a promising plant-based approach to removing heavy metals from the soil, and plant growth-promoting micro-organisms such as the fungi *Trichoderma* can enhance the ability of plants to accumulate metals. Inoculation of *Avena sativa* L. (oat) with *Trichoderma viride* enhances germination and seedling growth in the presence of Cd and, in this study, the growth of 6-month-old oat plants in Cd-contaminated soil was not increased by inoculation with *T. viride*, but a 1.7-fold increase in yield was observed. The content of Cd in oat shoots depended on the Cd content in the soil. Still, it was unaffected by the inoculation with *T. viride*. *A. sativa* metallothioneins (AsMTs) participate in plant–fungi interaction, however, their role in this study depended on MT type and Cd concentration. The inoculation of *A. sativa* with *T. viride* could be a promising approach to obtaining a high yield in Cd-contaminated soil without increasing the Cd content in the plant.

## 1. Introduction

Urbanisation, industrialisation, and agricultural activities increase heavy metal (HM) contamination in soil and water worldwide, resulting in increased accumulation of HM in plants and food [1]. Among various HMs, cadmium (Cd) is easily absorbed by plants. Due to the usage of phosphate fertilisers, sewage sludge, and atmospheric deposition, this toxic element is widely spread on agricultural land [2]. Cd causes plant cell damage through leaf rolling and chlorosis, and reduces root and shoot length and biomass [3,4,5]. This element has properties similar to the essential micronutrient zinc (Zn) and can compete with it in biological processes [6], leading to, e.g., oxidative stress and damage to photosystems [7]. The EU regulation 2023/915 sets the maximum levels of HM in food. For example, the maximum level of Cd for wheat germ is 0.2 mg kg^−1^, 0.05 mg kg^−1^ for barley and rye, and 0.1 mg kg^−1^ for other cereals, including oat [8]. For non-smokers, plant-based food is the primary route of Cd exposure. Multiple studies have shown higher amounts of Cd than regulatory threshold levels in edible plant parts, e.g., durum wheat [9] and rice [10]. Heavy metals have been persistent in the environment for centuries or even millennia. They can disperse to distant areas and accumulate in biotic and abiotic components of ecosystems. This is a potential threat to human health because HM can enter the food chain through bioaccumulation in the tissues of plants and animals [2]. Cadmium causes severe health problems, including congenital disabilities and osteomalacia, and affects the functioning of the kidneys, respiratory system, circulatory system, and central nervous system. The best-known example of Cd toxicity is Itai-Itai disease. This Cd poisoning occurs among inhabitants of the Jinzu River in Japan and is mainly characterised by severe pain as a result of osteomalacia [11]. Therefore, the remediation of Cd-contaminated arable land is essential for food security.

Plants have evolved several mechanisms to maintain the homeostasis of micronutrients and detoxify non-essential HMs, including metal transporters and metal-binding proteins, peptides, and low molecular weight ligands. Metallothioneins (MTs) are small proteins that maintain metal (Zn and Cu) homeostasis and detoxify hazardous metals (Cd) by binding metal ions via cysteine residues. In plants, four MT types (MT1-4) differ in the number and arrangement of the cysteines. Several lines of evidence suggest that different MT types fulfil different roles [12,13,14]. MTs can act as antioxidants due to the presence of the sulfhydryl groups of cysteine residues [3,15]. This could be one of the reasons why *MT* expression is activated in plants’ responses to various stress-inducing factors [3,16,17,18,19]. In silico analyses of promoters of *MT* genes in canola (*Brassica napus* L.), *Arabidopsis thaliana* (L.) Heynh., rice (*Oryza sativa* L.), maize (*Zea mays* L.), and oat (*Avena sativa* L.) showed the presence of *cis*-regulatory elements (CREs) involved in the response to light, phytohormones, drought, and other abiotic stresses [3,17,20,21,22]. Moreover, the expression of *MTs* can be affected by microorganisms. For example, *MT* expression was determined in *Festuca arundinacea* (Schreb.) Darbysh inoculated or not inoculated with the fungus *Epichloë coenophiala* and subjected to nickel (Ni) stress. In non-inoculated plants, the *MT* expression was higher and increased with an increase in Ni concentration. In inoculated samples, the level of MT was similar in most of the tested Ni concentrations [23].

In soil, microorganisms maintain ecological balance. They are responsible for up to 90% of all processes in soils; without them, the soil becomes lifeless [24]. They can interact with each other and other living organisms, including plants [25]. HM-degraded areas often have low micro-organism activity, and to restore the degraded soil, it is crucial to re-establish microorganism populations [26]. Fungi belonging to the genus *Trichoderma* are fast-growing and omnipresent in the environment, and they are found in soil, water, and air. Several species belonging to *Trichoderma* can promote plant growth and development; some of them have been shown to increase plant growth by up to 300% [27]. They can inhibit the growth of some fungal plant pathogens, e.g., *Botrytis cinerea*, *Colletotrichum* sp., and *Fusarium culmorum* [28]. *Trichoderma* spp. produce many secondary metabolites, including indole-3-acetic acid and other auxin analogues that promote the growth of plant roots [29]. Moreover, they secrete organic acids like citric acid, gluconic acid, and fumaric acid, reducing soil pH, which increases the bioavailability of soil macroelements such as phosphorus for plants [30]. They can also increase the uptake of microelements by plants via solubilisation of, e.g., Cu, Zn, manganese (Mn), and iron (Fe) [31,32]. By lowering the soil pH, *Trichoderma* can also increase the bioavailability of hazardous HM [33]. Fungi belonging to *Trichoderma* were found to be highly tolerant to high concentrations of various elements, including micronutrients like nickel (Ni), copper (Cu), and zinc (Zn), but also non-essential elements like lead (Pb) and arsenic (As) [34,35,36,37,38]. Therefore, it was proposed that *Trichoderma* could be used to increase the phytoextraction of HM. For example, *Brassica juncea* (L.) Czern. plants treated with *Trichoderma atroviride* F6 accumulated more Cd and Ni than non-inoculated plants [33]. *Trichoderma* also increased the uptake of Cd, chromium (Cr), Cu, Zn, and Ni by plants like *Miscanthus x giganteus* J.M. Greef, *Salix* sp. L., *Phalaris arundinacea* L., and *Panicum virgatum* L. [26]. Moreover, fungi belonging to *Trichoderma* can accumulate HM. For example, *Trichoderma viride* bioaccumulated Cd and Pb, and the bioaccumulation efficacy increased with the increasing HM metal concentration in the medium [39]. Interestingly, the biomass production of *Trichoderma simmonsii* (UTFC 10063) increased by 46.1% when the fungus was cultured in a medium containing Cd. Still, the bioaccumulation efficacy of Cd decreased with increased Cd concentration [40].

The interactions of saprophytic fungi *Trichoderma* with plants are widely described in the literature [41,42,43,44]. Oat (*Avena sativa* L.) belongs to mycorrhizal plants, and most of the research has been conducted on mycorrhizal fungi and their effects on oat growth and yield [45]. There is little data on the interactions of saprophytic fungi with oat. Therefore, the potential role of saprophytic fungi *T. viride* in promoting the growth of *A. sativa* in Cd-contaminated soils and the possible molecular mechanisms underneath these interactions were evaluated in this study. Oat is the world’s sixth most important food, feed, and industrial cereal [46]. The importance of oats in the human diet increases constantly [47]. Therefore, it is crucial to understand the mechanisms underlying the uptake, transport, and accumulation of HM in organs of this plant. In addition, the well-developed root system [48,49,50] and the ability to accumulate toxic HM, including Cd and Pb [51,52], make this plant potentially suitable for phytoremediation. This study aimed to assess the ability of *T. viride* to increase oat’s tolerance to Cd and, at the same time, increase Cd accumulation. Moreover, based on data from the literature, we hypothesised that oat MTs are involved in Cd detoxification, accumulation, and interaction with *T. viride*. Therefore, the in vivo metal-binding ability of oat MT1-4 was verified via heterologous expression in bacteria cells and *AsMTs* expression was investigated in the early stages of oat growth in Cd-contaminated soil. Our results suggest that inoculating oat seeds with *T. viride* could increase oat yield in Cd-contaminated soils without increasing Cd-accumulation in above-ground parts of plants.

## 2. Materials and Methods

### 2.1. Microorganisms

Six previously identified *T. viride* strains of known plant-promoting properties were used in this study (NCBI GenBank accession numbers: T1—OL221590.1, T2—OL221591.1, T3—OL221592.1, T4—OL221593.1, T5—OL221594.1, T6—OL221595.1) [53]. The fungi were grown in liquid potato dextrose media or on potato dextrose agar (PDA) (Biocorp, Warsaw, Poland) at 23 °C. The fungi were kept on PDA slants at 4 °C for stock culture.

### 2.2. Metal Resistance of T. viride and Minimal Inhibitory Concentration

Cu, Zn, and Cd ions were added to the PDA medium separately at increasing concentrations from 0 to 29.8 mM for Zn, 2.6 mM for Cu, and 3.7 mM for Cd. The PDA plates were then inoculated with a mycelial disk of 7 mm diameter and grown for 7 days at 23 °C. The Minimal Inhibitory Concentration (MIC) was defined as the lowest concentration of metal that wholly inhibited fungi growth [54]. The experiment was repeated three times.

### 2.3. Growth of A. sativa in the Presence of Fungi

Seeds of *Avena sativa* L. cultivar Bingo (Plant Breeding Strzelce Ltd., PBAI Group, Strzelce, Poland) were sterilised with a mixture of 30% hydrogen peroxide and 96% ethanol (1:1, *v:v*) for 1 min. The seeds were then rinsed six times with sterile distilled water. Sterile seeds were then suspended in *T. viride* T5 spore suspension. To obtain spore suspension, sterile distilled water was poured on the PDA plate with a one-week-old fungi culture, and spores were suspended using a cell spreader. The suspension was then filtered using sterile MiraCloth (Calbiochem, Merck, Darmstadt, Germany), and the number of spores in the filtrate was counted using a hemocytometer. The solution was diluted to the final concentrations of 10^6^, 10^4^_,_ and 10^2^ spores mL^−1^ and used on the same day. Sterile oat seeds were inoculated with spore-suspension by incubation for 15 min, with shaking at room temperature. The inoculated seeds were placed in glass Petri dishes lined with filter paper moistened with 3.5 mL of sterile distilled water. The control was non-inoculated seeds. The seeds were kept in darkness at 23 °C for six days. The germinated seeds were counted every day, and on the 6th day, the lengths and fresh and dry (moisture analyser MA 110.R, RADWAG, Radom, Poland) biomass of shoots and roots were measured. Germination parameters, i.e., germination percentage (G), germination index (GI), mean germination time (MGT), mean germination rate (MGR), and the coefficient velocity of germination (CVG) [55] were calculated according to the formulas provided in the cited literature. The experiment was repeated three times.

### 2.4. Effect of Heavy Metals on the Germination and Growth of A. sativa Seedlings

Seeds were prepared as described above, then placed on Petri dishes lined with filter paper moistened with 3.5 mL of sterile distilled water (control) or solutions of 25, 80, 150, or 245 µM Cd (as CdSO_4_ solution). The seeds were kept in darkness at 23 °C for six days. Every day, the number of germinated seeds was counted, and on the 6th day, the lengths and fresh and dry (Moisture analyser MA 110.R, RADWAG, Radom, Poland) biomass of shoots and roots were measured. The experiment was repeated three times.

### 2.5. Effect of T. viride on the Growth of A. sativa in the Presence of Heavy Metals

Seeds inoculated with *T. viride* T5 spore suspension (10^2^ spores mL^−1^) were placed on Petri dishes lined with filter papers moistened with 3.5 mL of sterile distilled water or solution of 25, 80, 150 or 245 µM Cd (as CdSO_4_ solution). Non-inoculated seeds served as a control. The seeds were kept in darkness at 23 °C for six days. Every day, the number of germinated seeds was counted, and on the 6th day, the lengths and fresh and dry biomass of shoots and roots were measured. The experiment was repeated three times.

### 2.6. Pot Experiment

For the pot experiment, a mixture of autoclaved soil and sand (5:1, *v*:*v*), amended with CdSO_4_ solution to the final concentration of 1, 5, 10, or 20 mg Cd kg^−1^ of soil, was used. To ensure the binding of Cd ions to the soil particles, the soil–Cd mixture was incubated for two weeks before seed sowing. Oat seeds were inoculated with 10^6^ spores mL^−1^ solution of *T. viride* T5, as described above, and 5 seeds per pot were sown (for each condition, 4 pots were used). The control was non-inoculated seeds. The plants were watered with tap water twice a week, and once a month, they were watered with Hoagland solution. After two weeks, leaves were collected and frozen in liquid nitrogen for gene expression analyses. After 6 months, shoot and root length and fresh and dry (moisture analyser MA 110.R, RADWAG, Radom, Poland) biomass were measured, and the number of leaves, seeds, and panicles was counted.

### 2.7. Level of Heavy Metals in A. sativa L. Plants

Dry shoot biomass was ground using a mortar and pestle, and the content of Cu, Cd, and Zn was analysed by ICP-MS 7500 CX (Agilent Technologies, Santa Clara, CA, USA) in the Instrumental Analysis Laboratory, Department of Chemistry, Nicolaus Copernicus University in Toruń. The analyses were performed in three biological replicates.

### 2.8. Identification of Metal-Responsive Elements in the Promoters of A. sativa Metallothioneins

A 1500 bp region upstream of the ATG codon for all *AsMT* genes was downloaded from the GrainGenes database (https://wheat.pw.usda.gov/, accessed on 20 April 2023). Metal response elements (MRE) and copper response elements (CuRE) were identified in the promoters of *A. sativa MT*s using the following sequences as well as their reverse and complementary sequences: 5′-TGCAGGC-3′ [56], 5′-TGCRCNC-3′ [56,57], 5′-TGCAACC-3′, 5′-TGCACCCC-3′, 5′-GAGAGCA-3′ [58] and 5′GTAC-3′ [59].

### 2.9. Functional Analysis of AsMT1-4 in E. coli

Expression constructs of *AsMT1-3* were prepared as described previously [16]. The coding region of *AsMT4* was amplified with sequence-specific primers containing restriction sites for *Nde*I in the forward primer 5′-AAACATATGGGCTGCGACGACAAGTG-3′ and *Xho*I in the reverse primer 5′-AACTCGAGTCAGGCGGTGGAG-3′. The PCR products were digested with *Nde*I and *Xho*I, and ligated into a pET21a(+) expression vector (Novagen, Darmstadt, Germany) to be later transformed into *E. coli* DH5α. The plasmids were isolated (Gene MATRIX Plasmid Miniprep DNA Purification Kit; EURx, Gdańsk, Poland) and sequenced to confirm the presence of the correct open reading frame (Genomed, Warsaw, Poland). The constructs were named pET-*AsMT1-4*.

For functional analysis, the *E. coli* Rosetta (DE3) cells (Novagen, Darmstadt, Germany) were transformed with an empty pET21a vector (control) or pET-*AsMT1-4* constructs using the heat shock method [53]. Overnight cultures of transformed bacterial cells were diluted (1:100, *v:v*) in LB medium with antibiotics (50 μg mL^−1^ ampicillin and 34 μg mL^−1^ chloramphenicol) to OD_600_ ≈ 0.2. To induce heavy metal stress, the cultures were supplemented with solutions of ZnSO_4_ or CdSO_4_ to final concentrations of 0.25 mM or 0.5 mM ZnSO_4_ and 0.1 mM or 0.25 mM CdSO_4_. The expression of *MT*s was induced using isopropyl-β-D-1-thiogalacto-pyranoside (IPTG) at the final concentration of 0.1 mM, avoiding high transgene overexpression. The controls were cultures without HM. The bacteria were incubated for 7 h (37 °C, 180 rpm), and OD_600_ (Implen OD_600_ DiluPhotometer, München, Germany) was measured every hour. The analysis was performed in three technical replicates for each of the three biological replicates. The growth rate of *E. coli* cultures was expressed as the slope of a linear proportion of the growth curve and was calculated using Microsoft Excel.

### 2.10. Gene Expression

Plant tissues were ground in liquid nitrogen, and 100 mg of the ground tissue was used for RNA isolation using an RNeasy kit (QIAGEN, Hilden, Germany). The quality and quantity of the isolated total RNA were checked via spectrophotometric measurement using a NanoDrop^TM^ Lite Spectrophotometer (Thermo Fisher Scientific, Waltham, MA, USA) and agarose gel electrophoresis stained with EtBr. The RNA was then treated with 1 U of DNase (Thermo Fisher Scientific, Waltham, MA, USA) to remove DNA contamination. The cDNA was synthesised from 1 µg of RNA using a mixture of 2.5 μM oligo(dT)20 primer and 0.2 μg of random hexamers with an NG dART RT Kit (EURx, Gdańsk, Poland), according to the manufacturer’s protocol. The reaction was performed at 25 °C for 10 min, followed by 50 min at 50 °C. The cDNA was stored at −20 °C.

The RT-qPCR reaction mixture included 4 μL of 1/30 diluted cDNA, 0.5 μM of gene-specific primers (Table 1), and 5 μL of LightCycler 480 SYBR Green I Master (Roche, Penzberg, Germany) for a total volume of 10 μL. Eukaryotic Initiation Factor 4A-3 (*EIF4A*) was a reference gene [60]. The reactions were performed in three technical replicates using LightCycler 480 Instrument II (Roche, Penzberg, Germany). The thermal cycling conditions were as follows: 95 °C for 5 min, 95 °C for 10 s, 60 °C for 20 s, and 72 °C for 20 s over 40 cycles. The SYBR Green I fluorescence signal was recorded at the end of the extension step in each cycle. The melt curve analysis confirmed the assay’s specificity, i.e., increasing the temperature from 55 to 95 °C at a ramp rate of 0.11 °C/s. The fold change in gene expression was calculated using LightCycler 480 Software, release 1.5.1.62 (Roche, Penzberg, Germany) [3,16,17].

### 2.11. Statistical Analysis

Statistical analyses were conducted using Microsoft Excel and RStudio [61]. The results are expressed as mean values with error bars representing standard error (SE). The one-way ANOVA (post hoc Tukey and Dunn’s tests), or the Kruskal–Wallis (post hoc Mann–Whitney test), were conducted based on sample type, normality, and homogeneity. Correlations were calculated using the Pearson correlation coefficient.

## 3. Results

### 3.1. Tolerance of T. viride to Cd, Cu, and Zn

The tolerance of six *T. viride* fungi to Zn, Cu, and Cd was tested using the minimal inhibitory concentration (MIC) method. MIC is defined as the lowest concentration of metal that completely inhibits fungi growth [54]. All six fungi could survive in tested metal concentrations (Table 2). The highest tolerance to Cd was observed for *Trichoderma* strain T1 (3.6 mM), but the same fungi had the lowest tolerance to Zn (22.3 mM). Strain T6 could tolerate Cu in concentrations lower than 2.5 mM, but strain T5 could not grow in 1.6 mM Cu. Based on these results, *T. viride* T5 was chosen for further experiments because it had a high tolerance to both Cd and Zn.

### 3.2. Seed Germination and Seedling Growth of A. sativa in the Presence of T. viride

To examine the effect of the inoculation of oat seeds with the spores of *T. viride* T5 on seed germination and early seedling growth, we inoculated oat seeds with fungal spores at concentrations 10^2^, 10^4^, and 10^6^ spores mL^−1^ (Table 3). The highest germination percentage, which reflects the viability of the seed population, was observed for seeds inoculated with 10^4^ spores. In contrast, treatment with 10^6^ spore concentrations significantly decreased germination percentage (G) compared to non-inoculated seeds. Inoculation of seeds with 10^4^ spores also increased the germination index (GI) (a measure of germination percentage and speed) and other tested parameters; however, the differences were not statistically significant. The highest GI was observed for 10^6^ spore concentration, i.e., a 1.1-fold significant increase. Moreover, mean germination time (MGT), mean germination rate (MGR), i.e., a reciprocal of MGT that measures the time it takes for the seed to germinate, and coefficient velocity of germination (CVG), which is an indicator of the rapidity of germination, were increased by the inoculation with spores at the concentration of 10^6^ (Table 3).

On the other hand, we observed that higher concentrations of *T. viride* spores (10^4^ and 10^6^) did not positively affect the growth of oat seedlings (Table 4). For the spore concentration 10^6^, shoot length and biomass were the same as control plants, but roots had 1.4- and 2.0 times lower fresh and dry biomass, respectively. For plants inoculated with spores at a concentration of 10^4^, the shoots were 1.2 times shorter, but the roots’ growth was unaffected compared to the control (Table 4). On the other hand, slight growth stimulation of oat seedlings was observed only when seeds were inoculated with 10^2^ spores, i.e., a 1.1-time increase in shoot dry biomass. Interestingly, a 1.4-time decrease in fresh root biomass after inoculation with spores at 10^2^ was also noticed (Table 4).

### 3.3. Effect of Cadmium and T. viride on the Seed Germination and Seedling Growth of A. sativa

Further, the effect of Cd and simultaneous Cd and *T. viride* T5 treatment on oat seed germination (Table 5) and seedling growth (Table 5) was tested. Cd treatment did not only affect the total number of germinated seeds (G)—all other parameters, reflecting the germination speed, were negatively affected by Cd treatment (Table 5). For example, GI was 1.2-fold and MGR 1.4-fold lower for seeds germinated in the presence of 245 μM Cd than the control. The inhibitory effect of Cd on germination was dependent on Cd concentration. After inoculation with *T. viride* spores, the negative impact of Cd on germination was also observed; however, the differences were not statistically significant (Table 5). Moreover, non-inoculated seeds in the presence of 150 and 245 µM Cd germinated more slowly (as shown by higher GI, MGR, and CVG, and lower MGT) than inoculated seeds. Interestingly, inoculation with *T. viride* spores decreased the total number of germinated seeds (Table 5).

Table 6 shows the morphological parameters of 6-day-old oat seedlings that grew in the presence of Cd and/or *T. viride* spores. The increase in Cd concentration decreased the length of shoots and roots, and fresh and dry biomass, of both inoculated and non-inoculated samples. The most noticeable decrease in shoot length was observed for plants treated with 150 µM Cd—1.8 and 1.6 times shorter for non-inoculated and inoculated samples, respectively, than the control. Root length was the most affected by 245 µM Cd, i.e., a 3.2 and 2.7 times reduction for non-inoculated and inoculated samples, respectively, compared to the control. Seedlings grown in the highest Cd concentration had the lowest fresh and dry shoot biomass in both non-inoculated and inoculated samples. The exception was the dry shoot biomass of non-inoculated plants, which was 1.1 times higher than in seedlings grown in control conditions. Inoculation with *T. viride* spores increased the growth of roots in high Cd concentrations, i.e., the length of roots was 1.2-fold higher in inoculated seedlings than in non-inoculated ones, both with 150 and 245 μM Cd (Table 6).

### 3.4. Effect of T. viride on the Growth and Yield of A. sativa Plants Grown in the Presence of Cd and on the Level of Cd Phytoextraction

**Figure 1:**
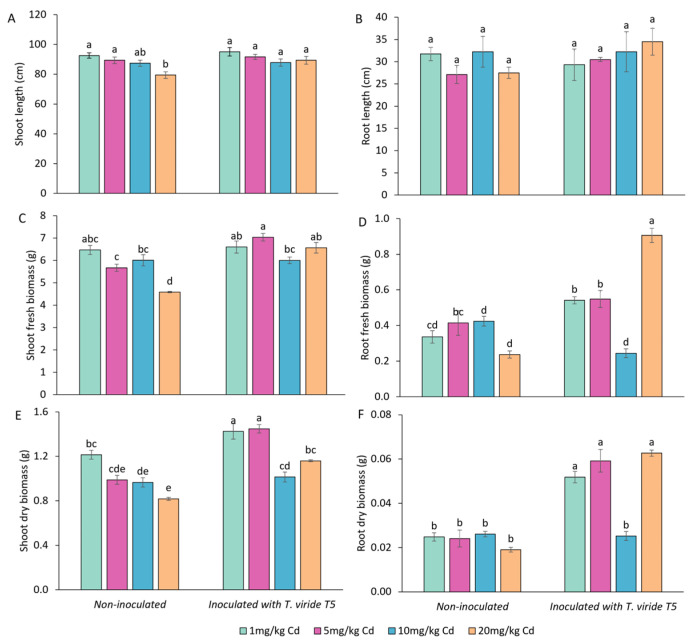
Effect of *Trichoderma viride* T5 inoculation on the growth of *Avena sativa* plants in soil containing 1 mg, 5 mg, 10 mg, and 20 mg of Cd per 1 kg of soil. Length, fresh and dry biomass of shoot (A, C, E, respectively) and root (B, D, F, respectively) were measured. Bars represent means (*n* = 40) ± SE. Means indicated with distinct letters are significantly different (Kruskal–Wallis, Dunn post hoc test, *p* < 0.05).

The following experiment was conducted to verify the effect of oat seed inoculation with *T. viride* T5 on mature plant growth and yield in Cd-contaminated soil. After six months of growth, the length of the oat roots was affected neither by inoculation with *T. viride* nor by cadmium treatment (Figure 1B). Fresh and dry root biomass in non-inoculated samples was not affected by cadmium. Inoculation caused an increase in fresh root biomass, i.e., in the presence of 1, 5, and 20 mg Cd kg^−1^ soil, fresh root biomass was respectively 1.6, 1.4, and 3.8 times higher when compared to non-inoculated samples (Figure 1D). Similarly, in inoculated plants grown in soil containing 1, 5, and 20 mg Cd kg^−1^, dry root biomass was respectively 2, 2.5, and 3.3 times higher when compared to non-inoculated samples (Figure 1F). In non-inoculated plants, the shoot length decreased with increased Cd concentration in soil—shoots of plants treated with 20 mg Cd were 1.2 times shorter than those treated with only 1 mg Cd. However, when inoculated with *T. viride* T5, the shoot length remained the same across all tested Cd concentrations (Figure 1A). Shoot fresh and dry biomass of non-inoculated plants decreased with increased Cd concentrations, with plants treated with 20 mg Cd having their fresh and dry biomass 1.4 and 1.5 times lower, respectively, compared to plants treated with only 1 mg Cd (Figure 1C,E). In inoculated samples, the fresh shoot biomass was similar across all Cd concentrations. Dry shoot biomass of inoculated plants treated with 1, 5, and 20 mg Cd was respectively 1.2, 1.5, and 1.4 times higher when compared to non-inoculated plants (Figure 1E).

Treatment with cadmium significantly affected the yield, i.e., the number of panicles was 1.7-fold lower, and the number of seeds was 2.2-fold lower in plants grown in soil containing 20 mg kg^−1^ of Cd compared to plants grown in soil containing 1 mg kg^−1^ of Cd (Table 7). Similar observations were made for plants treated with *T. viride* T5; however, for Cd concentration, the number of panicles and seeds was higher in inoculated plants compared to in non-inoculated ones. For example, plants inoculated with *T. viride* grown in soil containing 20 mg kg^−1^ of Cd produced 1.7-fold more seeds and 1.3-fold more panicles than non-inoculated plants grown in the same Cd concentration (Table 7).

To assess the potential of *A. sativa* for Cd phytoextraction and the potential involvement of *T. viride* in this process, Cu, Cd, and Zn content was analysed in the above-ground parts of 6-month-old plants (Table 8). As expected, the concentration of Cd in the oat shoots increased with an increase in Cd concentration in the soil, i.e., plants grown in soil with 20 mg kg^−1^ of Cd had 6.6 times (non-inoculated) and 5.3 times (inoculated) higher Cd concentration in shoots than plants grown in soil with 1 mg kg^−1^ of Cd. Inoculation with *T. viride* T5 spores did not significantly increase the Cd uptake by oat. The content of copper in oat shoots was affected neither by Cd concentration in soil nor by inoculation with *T. viride* T5 spores. Interestingly, the level of Zn in shoots of oat plants was the highest in plants growing in soil containing 20 mg Cd kg^−1^_,_ and it was 1.6 and 1.8 times higher in non-inoculated than in inoculated plants, respectively, compared to plants growing in soil contaminated with 1 mg Cd kg^−1^. Inoculation did not increase the Zn uptake regardless of Cd concentration in the soil (Table 8).

**Figure 2:**
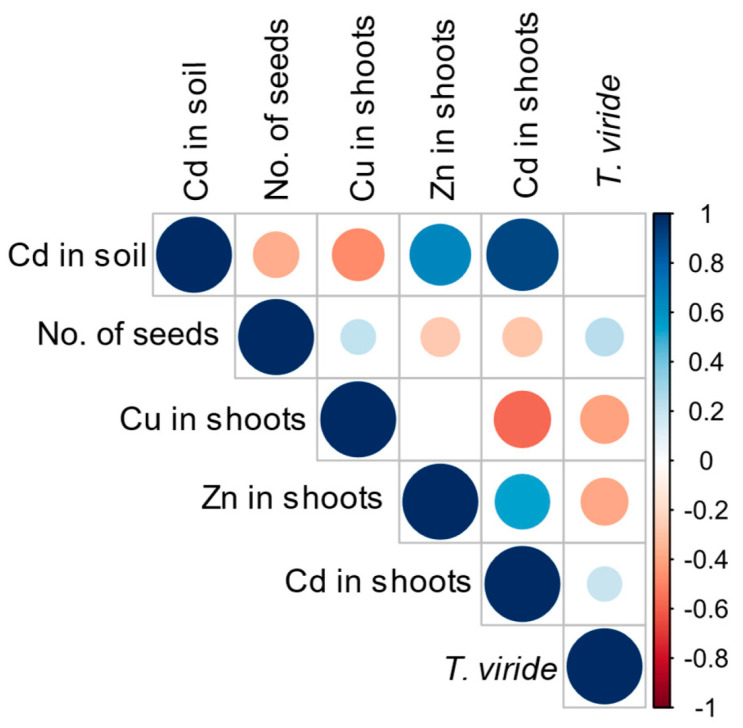
Pearson correlation between the amount of cadmium added to the soil, number of seeds, levels of Cu, Zn, and Cd in *Avena sativa* shoots, and the inoculation of oat seeds with *Trichoderma viride* T5 spores. Only significant correlations are shown.

Positive correlations were observed between the amount of cadmium added to the soil and the level of Zn and Cd in oat plants. In contrast, the level of Cd in soil was negatively correlated with the level of Cu in oat shoots (Figure 2). Cadmium application was also negatively correlated with shoot length, fresh and dry biomass, and number of panicles and seeds (Supplementary Figure S3). A positive correlation was observed between inoculation with *T. viride* T5 spores and root length, fresh and dry biomass, and shoots’ fresh and dry biomass (Supplementary Figure S3). Interestingly, a negative correlation between inoculation with *T. viride* T5 spores and the levels of Cu and Zn, and a positive between inoculation with *T. viride* spores and the level of Cd, were observed (Figure 2).

### 3.5. Functional Analysis of A. sativa Metallothioneins (AsMT1-4) in Bacteria Cells

**Figure 3:**
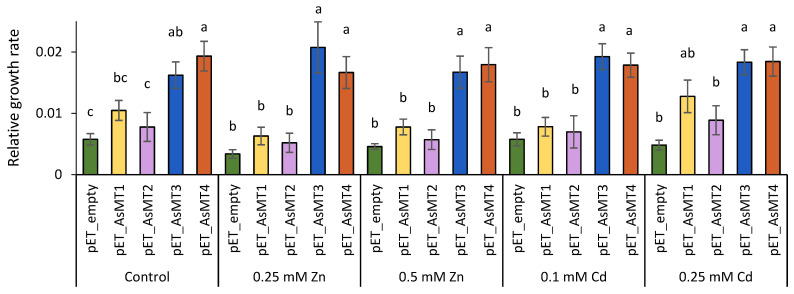
Comparison of the growth of *Escherichia coli* cells transformed with empty pET21a(+) vector and pET21a(+) vectors harbouring coding regions of *AsMT1-4* in LB medium (control) and LB medium supplemented with Zn or Cd ions. The expression of *AsMT1-4* was induced by 0.1 mM IPTG. The relative growth rate is expressed as a slope of bacterial growth curves obtained by plotting optical density against time. Bars represent means (*n* = 9) ± SE. The results obtained for a given condition were compared, and distinct letters indicate significant differences between *E. coli* carrying different plasmids (Kruskal–Wallis, Mann–Whitney; *p* < 0.05).

To verify the metal-binding ability of oat MT1-4, the proteins were expressed in *E. coli* cells in the presence of Zn and Cd (Figure 3). Bacteria transformed with plasmids carrying *AsMT3* and *AsMT4* grew faster under control and stress conditions caused by metal ions than bacteria transformed with an empty pET vector. The highest difference in growth rates between bacteria transformed with pET_AsMT3 and pET_AsMT4, and bacteria bearing an empty pET vector (6 and 5 times higher growth rates, respectively) was observed in medium supplemented with 0.25 mM Zn. The expression of *AsMT1* and *AsMT2* in bacterial cells did not increase bacteria growth. To verify the possible adverse effect of IPTG on bacteria growth, the experiment was also performed without the addition of IPTG (Supplementary Figure S1). Only bacteria transformed with pET_AsMT4 had a faster growth rate (both under control conditions and in the presence of Zn and Cd ions) than bacteria transformed with the empty pET vector.

### 3.6. Expression of A. sativa AsMT1-4 in Plants Growing in Cd-Contaminated Soil

**Figure 4:**
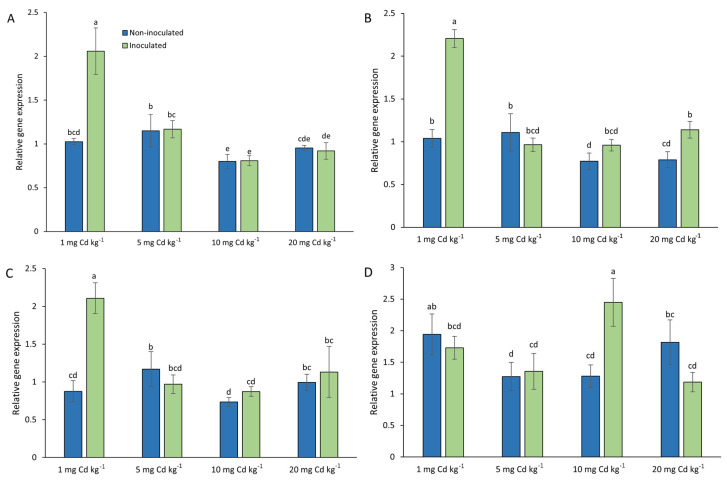
Relative gene expression of *Avena sativa* metallothioneins (**A**) *A*s*MT1*, (**B**) *A*s*MT2*, (**C**) *A*s*MT3*, and (**D**) *A*s*MT4* in two-week-old oat seedlings, inoculated (green bars) or non-inoculated (blue bars) with *Trichoderma viride* T5, grown in soil containing 1, 5, 10, and 20 mg Cd per kg of soil. Bars represent means (*n* = 2) ± SE. Distinct letters mark significant differences (one-way ANOVA, Tukey post hoc test, *p* < 0.05).

To give insight into molecular mechanisms underlying Cd detoxification and interaction with *T. viride* in oat plants, gene expression of *AsMT1-4* was analysed (Figure 4). The possibility that heavy metals induce the expression of oat MTs was verified by in silico analysis of promoter regions of *AsMT1-4* genes (Supplementary Table S1). In total, 4, 4, 8, and 11 MRE were found in *AsMT1*, *AsMT2*, *AsMT3*, and *AsMT4* promoters, respectively. The most common motif was the CuRE motif 5′-GTAC-3′, which appeared 20 times in the promoter sequences of *AsMT1-4.* The second most abundant motif was 5′-TGCRCNC-3′, found five times (Supplementary Table S1).

In inoculated plants, the expression of *AsMT1*, *AsMT2*, and *AsMT3* in the presence of 1 mg Cd was over two times higher than in non-inoculated samples. However, in plants growing in soil containing 5, 10, and 20 mg of Cd per kg of soil, the *AsMT1-3* expression in both variants, i.e., non-inoculated and inoculated, was on a similar level. The exception was *AsMT2*, where the expression in inoculated seedlings growing in 20 mg/kg of Cd was 1.5 times higher than in non-inoculated plants. The expression of *AsMT4* was the highest in samples inoculated with *T. viride* in seedlings grown in the presence of 10 mg/kg of Cd (almost 2-fold higher than in non-inoculated plants). In other variants, the *AsMT4* expression in both inoculated and non-inoculated samples was comparable (Figure 4).

Correlation analyses showed high positive correlations among *AsMT1-3* but not between *AsMT1-3* and *AsMT4* (Supplementary Figure S4). Positive correlations were also observed between *T. viride* inoculation and expression of *AsMT1-3*, but negative correlations were noted between *AsMT1-3* expression and the level of Cd in soil. Neither the inoculation with *T. viride* nor the level of Cd in the soil was correlated with the expression of *AsMT4* (Supplementary Figure S4).

## 4. Discussion

Anthropogenic activities, like mining, the metallurgic industry, fossil fuel extraction, global transport, and agriculture, contribute to the increasing concentration of heavy metals in soil [62,63]. Even low concentrations of HM can become hazardous since they accumulate in the food chain [64,65]. Thus, it is essential to ensure that HM levels in soils and crops meet regulatory standards [66,67]. Since soil worldwide is contaminated with HM, there is an urgent need for practical, eco-friendly, and cost-effective remediation methods [68]. Phytoremediation is an eco-friendly and cost-effective method of removing hazardous pollutants, including HM, by plants. The effectiveness of this method can be increased by applying microorganisms that can interact with plants to counteract stressful environmental conditions and improve the plant’s capacity to absorb pollutants. Understanding how microorganisms and plants respond to HM in their environment is crucial for developing this remediation method [69,70]. Among several microorganisms, fungi belonging to *Trichoderma* are considered suitable for phytoremediation due to their ability to use various materials as a carbon source, including plastics [71,72], their ability to promote plant growth and development [18,73,74,75], and their resistance to xenobiotics [76]. Analysis of the *Trichoderma harzianum* transcriptome in response to Cd treatment revealed the up-regulation of cellular homeostasis, vesicle-mediated transport, and RNA processing. Moreover, sulfur-compound biosynthesis and glutathione metabolism were induced [77]. *T. viride* strains tested in this study differed in Cu, Cd, and Zn tolerance. Strain T1 exhibited the highest tolerance towards Cd and the lowest towards Zn, and the opposite was observed for strain T6. The tolerance of *Trichoderma* to HM, as reported in the literature, is variable. For example, for Zn, the concentration that inhibited the growth was reported to be four mM for unclassified *Trichoderma* strains [78] and 11.47 mM for *Trichoderma atrioviride* [35]. Those values range from 1.8 mM [79] to 2.67 mM [35] for Cd. This comparison showed that the strains of *T. viride* analysed in this study were highly tolerant to Zn, whereas tolerance to Cd was similar to other *Trichoderma* species and isolates. In contrast, the tolerance to Cu of *T. viride* T1-6 was relatively low since *T. harzianum* and *T. virens* tolerated Cu up to 12 mM [35,80]. Due to the similar physicochemical properties of Zn and Cd, the *T. viride* strain T5 was selected for further experiments because this stain had a high tolerance to both cadmium and zinc.

A seed coat is a rigid structure that protects the embryo from soil pollutants, including HM. During germination, it ruptures, and Cd content increases in seeds [81]. The negative impact of Cd on germination was shown for bean (*Phaseolus vulgaris* L.) [81], *Sorghum bicolor* (L.) Moench [82], and wheat (*Triticum aestivum* L.) [83]. The amount of Cd needed to inhibit germination differs from species to species and within one species from cultivar to cultivar [84]. Interestingly, it was also shown that low levels of Cd might positively affect seed germination and seedling growth [85]. In this study, Cd inhibited oat seed germination and further seedling growth, and the negative effect was more substantial for higher cadmium concentrations. In uncontaminated soil, the mean value of Cd is 0.36 mg/kg, although the Cd concentrations greatly depend on continent, country, and soil types [86]. The mean concentration of Cd in European agricultural soil is 0.15 mg/kg; in the wide-ranging analysis, croplands containing as much as 52.99 mg/kg of Cd were detected [87]. The EU risk assessment predicted no effective Cd concentration in soil of 1.1 mg per kg of dry soil based on the toxicity for plants, invertebrates, and animals [87]. Therefore, this study used soil containing 1 mg/kg Cd as a control. The yield (as shown by the number of panicles and seeds) of oat plants was significantly decreased by cadmium. For example, plants grown in soil containing 20 mg/kg Cd produced less than half of the seeds produced by plants grown in the presence of 1 mg/kg Cd. Crop plants significantly differ in their tolerance to Cd contamination, and there are also substantial differences in Cd tolerance among cultivars of the same species. A significant decrease in rice yield in soil contaminated with 1 mg/kg and 3 mg/kg of Cd was observed; however, the number of seeds produced also depends on the tested cultivar [88]. Compared to other grasses, oat is relatively tolerant to Cd stress [89]. Similar to our observations, Cd in soil up to 25 mg/kg did not significantly affect the growth of oat plants, but the yield was reduced [90].

Fungi belonging to *Trichoderma* are known for their plant growth-promoting properties [28,42,44,74,75]. Our previous studies show that *T. viride* can promote the growth of *B. napus* [28,74]. Barley plants inoculated with *Trichoderma* have up to 20% higher dry biomass than non-inoculated plants [43]. Similar reports are available for rice [91], sunflower [92], and maize [93]. The positive effect of microbial inoculation is often visible only in stress conditions [94]. For example, in control conditions, inoculation with *Trichoderma* did not improve wheat growth. Still, under severe water stress, inoculated wheat plants had higher dry biomass, downregulated water stress-related genes, and lower levels of proline, hydrogen peroxide, and malondialdehyde compared to non-inoculated plants [95]. In this study, seed germination and seedling growth were not improved by inoculation with *T. viride* in control conditions. Still, in the presence of cadmium, inoculated seeds germinated quicker, and the growth of seedling roots was enhanced in the presence of 150 and 254 μM Cd. The growth of plants was improved in soil containing 20 mg/kg Cd by inoculation with *T. viride*. The most significant effect was the increase in yield by *T. viride* inoculation observed in all Cd concentrations. The improved growth of plants in the presence of cadmium by inoculation with *T. harzianum* [96] and *T. atrioviride* [33] was shown for *B. juncea*. Plant growth promotion by fungi in HM-contaminated environments may be caused by increasing root absorption area and nutrient uptake [33,96]. The growth of *Cicer arietinum* was enhanced by inoculation with *Trichoderma* sp.; however, the effect was more substantial when plants were co-inoculated with *Trichoderma* sp. and *Pseudomonas fluorescence* [97]. The authors further highlighted that mechanisms that allow micro-organisms to adapt to and survive in HM-contaminated environments include binding HM to the cell wall and using siderophores to stop the HM from entering the cell, metalloproteases that bind and sequester HM in the cell, efflux pumps that eliminate HM from the cells, and antioxidant systems that reduce the negative effect of HM [97]. Combined with the growth-promoting properties of fungi belonging to *Trichoderma* (i.e., production of auxin analogues, organic acids, and siderophores), an increased tolerance to HM stress in inoculated plants was observed [29,30,96,97]. Interestingly, the inoculation with *T. harzianum* did not improve the growth of barley [98], and inoculation with *Trichoderma* sp. did not increase the growth and yield [90] in Cd-contaminated soil. Our recent study demonstrated a significant increase in *B. napus* yield by inoculation with *T. viride* in a field experiment [73]. Those results indicate that the improvement of plant growth and yield depends on fungi species/strain, plant species/cultivar, and the condition of the experiments.

Some crop plants are considered Cd-hyperaccumulators, e.g., several species belonging to *Brassicaceae*, some legumes, and some cereals [99]. For example, *B. juncea* accumulated more than 400 μg/g dry weight in leaves [100] and wheat up to 18 mg/kg dry weight [101]. Interestingly, Cd tolerance and accumulation are not usually related [99]. For example, wheat Cd-sensitive cultivars accumulated more Cd than Cd-tolerant ones [102]. Also, for oat, low and high Cd-accumulating cultivars were described [103]. The amount of Cd accumulated in crops and the location of Cd within plants are crucial in terms of nutrition. The World Health Organization recommends consuming no more than 25 μg of Cd monthly per kg of body weight. Oat can survive in soil polluted with heavy metals by extracting the metals from it and transferring them to above-ground parts [52,89,90,104]. The increased concentration of Cd in the soil led to an increased concentration of Cd in oat shoots. It is a widely observed phenomenon that the application of fungi belonging to *Trichoderma* increases the amount of extracted heavy metals, and this effect was observed for Cd but not for Cu and Zn in this study. In a study by Cao et al. [33], *B. juncea* inoculated with *T. atroviride* extracted 24% more Ni and 8% more Cd from the soil than non-inoculated plants did. Applying *Trichoderma* increased Cd content in shoots of maize plants growing in a Cd-contaminated soil by 38%, compared to non-inoculated plants [38]. A study showed that applying *T. harzianum* positively affected Cd uptake in barley *(H. vulgare* L.) [98]. The application of *Trichoderma* improved the solubility of heavy metals and, as a result, increased their uptake by *Miscanthus x giganteus* J.M. Greef, *Panicum virgatum* L., *Phalaris arundinacea* L., and *Salix* sp. [26]. *A. sativa* has excellent potential to be used in phytoremediation since it can grow on low-quality soil, can tolerate higher concentrations of toxic metal ions, and has higher biomass than hyperaccumulators, making the whole process more efficient [89]. Cadmium uptake influences the uptake of micronutrients since Cd enters the root using micronutrient transporters such as transporters belonging to ZIP (zinc-regulated, iron-regulated transporter-like protein) [105,106] and NRAMP (natural resistance-associated macrophage protein) [107]. In this study, the increased Cd content in the shoots was positively correlated with the Zn content in the shoots but negatively with the content of Cu. Zn and Cd have similar physicochemical properties, and usually, the higher the Cd concentration in the soil, the lower the Zn content in plants [108,109]. However, various external factors, such as pH, affect the interplay between Cd and micronutrients in soil [110]. Moreover, within plants, Cd interacts with micronutrients. For example, high zinc reduces Cd transport to shoots, whereas, in low Zn conditions, zinc translocation to shoots is increased by Cd [111].

Metallothioneins’ primary and firmly documented role in all living organisms is the homeostasis of micronutrients, mainly zinc and copper, and the detoxification of toxic metals, mostly cadmium [112]. In mammals, the expression of *MT*s in response to heavy metals is regulated by transcription factor MTF-1 (MRE-binding transcription factor-1) that binds to conserved regulatory motif MRE (metal response element) 5′-TGCRCNC-3′ present in *MT* promoter sequences. MTF-1 contains zinc finger domains and recognises MRE upon Zn metallation [113]. In yeast, the expression of metallothionein *CUP1* is regulated by copper-sensing transcription factor ACE1 that binds to regulator element 5′-HTHNNGCTGD-3′ [114]. Relatively little is known about the molecular mechanisms underlying the induction of the expression of *MTs* by cadmium; however, it was demonstrated that Cd induces the expression of *MT* in animals [115], plants [116], and bacteria [117]. Databases used for the prediction of regulatory elements in plant-promoter sequences (e.g., PlantCARE [118] and New PLACE [119]) lack plant-specific MREs; therefore, the animal MRE consensus sequence was used. The potential role of the regulatory motifs similar to animal MREs in the induction of the expression of plant MTs in response to heavy metals has been confirmed [56,58,120]. In addition, plant-specific MRE [56] and CuRE (copper response element) identified in *Chlamydomonas reinhardtii* [121] were used. Multiple potential regulatory elements involved in response to biotic factors [3] and heavy metals present in analysed promoters support the hypothesis that AsMTs are engaged in plant interaction with *T. viride* and/or Cd response. Previously, we identified multiple abiotic stress response elements in *AsMT* promoters [3], and the role of AsMTs in response to drought [17] and osmotic [16] stress was demonstrated. Moreover, we suggested the role of AsMT1 and AsMT3 in Cd detoxification and the role of AsMT4 as a Zn specificity filter [3]. Heterologous expression of *AsMT3* and *AsMT4* in *E. coli* cells improved bacterial growth in the presence of Zn and Cd. Previously, it was shown that the expression of *Brassica rapa* L. *MT* types 1-3 increases the yeast tolerance to Zn, Cd, and Pb [122]. In contrast, the expression of *B. napus MT4* in *E. coli* cells improved the growth of bacteria in the presence of Zn, decreased it in the presence of Cu, and had no effect on its growth in the presence of Cd. The positive/negative impact on bacteria growth also depended on the concentration of metals and/or IPTG [123]. Metallothioneins were shown to be involved in HM hyperaccumulation. For example, in a model plant for hyperaccumulators *Thlaspi caerulescens* (J. Presl & C. Presl) F.K. Mey., the expression of *MT1* and *MT2* was higher than in closely related *Arabidopsis thaliana* (L.) Heynh. [124]. Moreover, the expression of *T. caerulescens MT3* was higher in a population with high Cd-accumulation and -tolerance [125,126]. A limited amount of evidence also suggests that MTs are involved in the interaction between plants and microorganisms. For instance, the expression of canola MTs types 1-3 was higher in seedlings inoculated with plant growth-promoting fungi *T. viride*. However, the enhanced expression was observed only for some *T. viride* strains, whereas for others, the *MT1-3* expression was unaffected [73]. On the other hand, inoculation of *B. napus* with spores of arbuscular mycorrhizal (AM) fungi increased the expression of *BnMT2* when plants were grown in soil without indigenous micro-organisms. In contrast, when indigenous microbes were present, the inoculation with AM spores decreased the expression of *BnMT2* [18]. Inoculation of willow (*Salix viminalis* L.), growing in heavy metal-contaminated soil with rhizosphere bacteria *Bacillus cereus*, increased the expression of *MT1*. No increase in *MT1* expression was observed after inoculation with the fungi *Hebeloma mesophaeum* [127]. In this study, Cd did not affect *AsMT* expression, but the inoculation with *T. viride* increased the expression of *AsMT1-3* in soil containing 1 mg/kg Cd and *AsMT4* in soil containing 10 mg/kg Cd. The response of MTs to metals depends not only on metal, plant species, and type of MT but also on plant organs and the amount of metal [128].

## 5. Conclusions

Inoculating *A. sativa* with *T. viride* might be a promising approach to increase the yield in Cd-contaminated soil without increasing the Cd content in plant tissues. Using *Trichoderma* in agriculture may improve the quantity and quality of produced food. This could be due to the fungi’s ability to produce auxin analogues, thus improving plant root growth. Moreover, the secretion of organic acids and siderophores affects the bioavailability of micronutrients, and toxic HM could be bound to fungi cell walls. This is of great importance because it could limit the inclusion of Cd in the food chain and thus improve the health of animals and humans. Meat consumption worldwide is declining for financial, environmental, and ethical reasons, thus, the importance of crops such as oats in nutrition is constantly increasing. There is an urgent need to develop strategies to enhance crop yields in contaminated soils without reducing food quality.

## Figures and Tables

**Table 1:** Sequence of primers used in this study.

Primer Name	Sequence 5′→3′	Target	Reference
AsMT1_qPCR_f AsMT1_qPCR_r	CAAACTGCAAGTGCGGGAAG TTGTTCTCATGAGCCACGCC	*AsMT1*	[17]
AsMT2_qPCR_f AsMT2_qPCR_r	CTGCGGAGGGTGCAAGATG AACGATGGCTTGGAAGAGGG	*AsMT2*	
AsMT3_qPCR_f AsMT3_qPCR_r	TCCACCATGTCGAACACCTG TGGCTCTTCTCGGTGTCAAC	*AsMT3*	
AsMT4_qPCR_f	CACGTGCGGAGAGCACTG	*AsMT4*	[3]
AsMT4_qPCR_r	ACAGGAGGCGCAGTCACAG		
EIF4A_f EIF4A_r	TCTCGCAGGATACGGATGTCG TCCATCGCATTGGTCGCTCT	*EIF 4A*	[60]

**Table 2:** Minimal inhibitory concentration (MIC) of Zn, Cu, and Cd for *Trichoderma viride* strains T1–T6.

*T. viride* Strain	Minimal Inhibitory Concentration [mM]		
	Zn	Cu	Cd
T1	22.3	1.7	3.6
T2	29.0	2.0	2.6
T3	28.5	2.4	2.6
T4	28.5	2.0	2.7
T5	29.2	1.6	2.9
T6	30.0	2.0	1.9

**Table 3:** Germination parameters of *Avena sativa* seeds treated with increasing concentration of *Trichoderma viride* T5 spores (10^2^, 10^4^_,_ and 10^6^ spores mL^−1^). Values are means (*n* = 100) ± SE. Values marked by distinct letters in a row differ significantly (one-way ANOVA, Tukey post-hoc test, *p* < 0.05).

*T. viride* T5 Spore Concentration (Spores mL^−1^)	G (%)	GI (days)	MGT (days)	MGR (1/days)	CVG
0 (Control)	93.45 ± 1.48 ^a^	5.10 ± 0.08 ^b^	1.90 ± 0.08 ^a^	0.55 ± 0.02 ^b^	54.98 ± 2.13 ^b^
10^2^	94.33 ± 1.11 ^a^	5.16 ± 0.05 ^ab^	1.84 ± 0.05 ^ab^	0.55 ± 0.01 ^ab^	55.42 ± 1.28 ^b^
10^4^	100.00 ± 0.20 ^a^	5.38 ± 0.04 ^ab^	1.62 ± 0.04 ^ab^	0.62 ± 0.01 ^ab^	61.76 ± 1.39 ^ab^
10^6^	80.00 ± 1.67 ^b^	5.63 ± 0.06 ^a^	1.38 ± 0.06 ^b^	0.73 ± 0.03 ^a^	73.33 ± 3.14 ^a^

G—germination percentage, GI—germination index, MGT—mean germination time, MGR—mean germination rate, CVG—coefficient velocity of germination.

**Table 4:** Effect of *Trichoderma viride* T5 inoculation with different spore concentrations (10^2^, 10^4^, and 10^6^ spores mL^−1^) on the growth of 6-day-old *Avena sativa* seedlings. Values are means (*n* = 40) ± SE. Values marked by distinct letters in a column differ significantly (one-way ANOVA, Tukey post-hoc test, *p* < 0.05).

Treatment	Spore conc.	Shoot Length (cm)	Fresh Shoot Biomass (g)	Dry Shoot Biomass (g)	Root Length (cm)	Fresh Root Biomass (g)	Dry Root Biomass (g)
Control (non-inoculated)	0	5.73 ± 0.28 ^a^	0.068 ± 0.004 ^a^	0.0054 ± 0.0005 ^ab^	8.26 ± 0.51 ^a^	0.060 ± 0.003 ^a^	0.0052 ± 0.0002 ^a^
Inoculated with *T. viride* T5	10^2^	5.24 ± 0.15 ^ab^	0.063 ± 0.003 ^a^	0.0062 ± 0.0002 ^a^	8.18 ± 0.49 ^a^	0.044 ± 0.003 ^b^	0.0052 ± 0.0002 ^a^
	10^4^	4.92 ± 0.23 ^b^	0.059 ± 0.004 ^a^	0.0051 ± 0.0005 ^ab^	7.74 ± 0.62 ^a^	0.052 ± 0.004 ^ab^	0.0048 ± 0.0003 ^a^
	10^6^	5.43 ± 0.13 ^ab^	0.061 ± 0.002 ^a^	0.0046 ± 0.0004 ^b^	7.69 ± 0.28 ^a^	0.043 ± 0.003 ^b^	0.0026 ± 0.0002 ^b^

**Table 5:** Germination parameters of *Avena sativa* seeds germinated in the presence of Cd (25–245 μM) or in water (0 μM). Seeds were inoculated with *Trichoderma viride* T5 spores at a concentration of 10^2^ mL^−1^ before sowing; control seeds were not inoculated. Values are means (*n* = 100) ± SE. Values marked by distinct letters in a column differ significantly (one-way ANOVA, Tukey post-hoc test, *p* < 0.05).

	Cd conc. [µM]	G (%)	GI (days)	MGT (days)	MGR (1/day)	CVG
Control (non-inoculated)	0	93.45 ± 1.48 ^a^	5.10 ± 0.08 ^a^	1.90 ± 0.08 ^b^	0.55 ± 0.02 ^a^	54.98 ± 2.13 ^a^
	25	88.75 ± 1.56 ^a^	4.88 ± 0.04 ^ab^	2.12 ± 0.04 ^ab^	0.47 ± 0.01 ^ab^	47.49 ± 0.74 ^ab^
	80	90.47 ± 1.11 ^a^	4.90 ± 0.05 ^ab^	2.10 ± 0.05 ^ab^	0.48 ± 0.01 ^ab^	48.25 ± 1.15 ^ab^
	150	92.38 ± 0.84 ^a^	4.52 ± 0.04 ^b^	2.48 ± 0.04 ^a^	0.41 ± 0.01 ^b^	40.71 ± 0.73 ^b^
	245	89.43 ± 2.15 ^a^	4.42 ± 0.05 ^b^	2.58 ± 0.05 ^a^	0.39 ± 0.01 ^b^	39.16 ± 0.81 ^b^
Inoculated with *T. viride* T5	0	94.33 ± 1.11 ^a^	5.16 ± 0.05 ^a^	1.84 ± 0.05 ^b^	0.55 ± 0.01 ^a^	55.42 ± 1.28 ^a^
	25	83.81 ± 1.37 ^a^	4.88 ± 0.03 ^ab^	2.12 ± 0.03 ^ab^	0.47 ± 0.01 ^ab^	47.36 ± 0.66 ^ab^
	80	89.05 ± 1.39 ^a^	4.91 ± 0.04 ^ab^	2.09 ± 0.04 ^ab^	0.48 ± 0.01 ^ab^	48.25 ± 0.98 ^ab^
	150	87.73 ± 0.76 ^a^	4.88 ± 0.05 ^ab^	2.12 ± 0.05 ^ab^	0.48 ± 0.01 ^ab^	47.90 ± 1.25 ^ab^
	245	86.85 ± 1.66 ^a^	4.79 ± 0.09 ^ab^	2.21 ± 0.09 ^ab^	0.48 ± 0.02 ^ab^	47.53 ± 2.09 ^ab^

G—germination percentage, GI—germination index, MGT—mean germination time, MGR—mean germination rate, CVG—coefficient velocity of germination.

**Table 6:** Effect of Cd (25–245 μM) and *Trichoderma viride* T5 inoculation (10^2^ spores mL^−1^) on the growth of 6-day-old *Avena sativa* seedlings. Values are means (*n* = 40) ± SE. Values marked by distinct letters in a column differ significantly (one-way ANOVA, Tukey post-hoc test, *p* < 0.05).

	Cd conc. [µM]	Shoot Length (cm)	Fresh Shoot Biomass (g)	Dry Shoot Biomass (g)	Root Length (cm)	Fresh Root Biomass (g)	Dry Root Biomass (g)
Control (non-inoculated)	0	5.73 ± 0.28 ^a^	0.068 ± 0.004 ^a^	0.0054 ± 0.0005 ^bc^	8.26 ± 0.51 ^a^	0.060 ± 0.003 ^a^	0.0052 ± 0.0002 ^b^
	25	4.27 ± 0.34 ^b^	0.052 ± 0.003 ^b^	0.0060 ± 0.0004 ^b^	6.47 ± 0.62 ^b^	0.047 ± 0.005 ^abc^	0.0052 ± 0.0004 ^b^
	80	5.60 ± 0.22 ^a^	0.066 ± 0.003 ^a^	0.0073 ± 0.0003 ^a^	6.46 ± 0.24 ^b^	0.058 ± 0.003 ^ab^	0.0065 ± 0.0003 ^a^
	150	3.21 ± 0.20 ^c^	0.047 ± 0.003 ^bc^	0.0042 ± 0.0001 ^d^	3.27 ± 0.19 ^cd^	0.037 ± 0.003 ^cd^	0.0030 ± 0.0000 ^d^
	245	3.85 ± 0.18 ^bc^	0.047 ± 0.003 ^bc^	0.0060 ± 0.0004 ^b^	2.57 ± 0.13 ^d^	0.028 ± 0.002 ^d^	0.0037 ± 0.0003 ^cd^
Inoculated with *T. viride* T5	0	5.24 ± 0.15 ^a^	0.063 ± 0.003 ^a^	0.0062 ± 0.0002 ^ab^	8.18 ± 0.49 ^a^	0.044 ± 0.003 ^bcd^	0.0052 ± 0.0002 ^b^
	25	3.42 ± 0.17 ^c^	0.045 ± 0.002 ^c^	0.0042 ± 0.0002 ^d^	4.59 ± 0.28 ^c^	0.032 ± 0.002 ^cd^	0.0037 ± 0.0001 ^cd^
	80	3.45 ± 0.21 ^c^	0.041 ± 0.003 ^c^	0.0044 ± 0.0001 ^cd^	4.32 ± 0.30 ^c^	0.034 ± 0.002 ^cd^	0.0041 ± 0.0002 ^c^
	150	3.28 ± 0.19 ^c^	0.042 ± 0.003 ^c^	0.0044 ± 0.0002 ^d^	3.75 ± 0.21 ^cd^	0.033 ± 0.002 ^cd^	0.0040 ± 0.0001 ^c^
	245	3.45 ± 0.22 ^c^	0.039 ± 0.003 ^c^	0.0042 ± 0.0003 ^d^	3.09 ± 0.20 ^d^	0.034 ± 0.009 ^cd^	0.0034 ± 0.0002 ^cd^

**Table 7:** The number of *Avena sativa* leaves, panicles, and seeds per plant grown in soil contaminated with Cd and inoculated with *Trichoderma viride* T5. Values are means (*n* = 15) ± SE. Values indicated with distinct letters in a column differ significantly (Kruskal–Wallis, Dunn post hoc test, *p* < 0.05).

	Cd Content in the Soil [mg kg^−1^]	Leaves Number	Panicles Number	Seeds Number
Non-inoculated	1	17.9 ± 1.1 ^b^	3.2 ± 0.4 ^bc^	11.9 ± 1.4 ^abd^
	5	17.1 ± 1.0 ^b^	2.7 ± 0.2 ^c^	10.0 ± 0.9 ^bd^
	10	15.4 ± 1.1 ^b^	1.8 ± 0.2 ^d^	6.5 ± 1.3 ^c^
	20	17.1 ± 0.7 ^b^	1.9 ± 0.4 ^cd^	5.5 ± 1.1 ^c^
Inoculated with *T. viride* T5	1	19.8 ± 0.8 ^a^	4.1 ± 0.4 ^b^	14.5 ± 1.3 ^ab^
	5	17.9 ± 0.8 ^ab^	4.2 ± 0.3 ^ab^	16.0 ± 1.5 ^a^
	10	16.5 ± 0.7 ^b^	2.2 ± 0.4 ^cd^	7.8 ± 1.4 ^cd^
	20	16.7 ± 0.8 ^b^	2.5 ± 0.5 ^cd^	9.5 ± 1.9 ^cd^

**Table 8:** Content of Cd, Cu, and Zn in shoots of *Avena sativa* plants inoculated or non-inoculated with *Trichoderma viride* T5 grown in soil contaminated with Cd. Values are means (*n* = 15) ± SE. Values indicated with distinct letters in a column are significantly different (one-way ANOVA, Tukey post hoc test, *p* < 0.05).

Treatment	Cd content in the Soil [mg kg^−1^]	Content in Shoots		
		Cd [mg kg^−1^]	Cu [mg kg^−1^]	Zn [mg kg^−1^]
Non-inoculated	1	0.143 ± 0.01 ^c^	5.674 ± 0.84 ^a^	29.625 ± 3.31 ^bc^
	5	0.209 ± 0.04 ^c^	5.751 ± 0.33 ^a^	49.310 ± 1.97 ^a^
	10	0.387 ± 0.07 ^bc^	4.249 ± 0.27 ^a^	39.665 ± 0.41 ^ab^
	20	0.931 ± 0.11 ^a^	4.048 ± 1.55 ^a^	47.805 ± 6.13 ^a^
Inoculated with *T. viride* T5	1	0.198 ± 0.03 ^c^	4.134 ± 0.94 ^a^	25.515 ± 4.16 ^c^
	5	0.377 ± 0.07 ^bc^	5.145 ± 1.60 ^a^	36.770 ± 8.36 ^abc^
	10	0.577 ± 0.08 ^b^	3.618 ± 1.26 ^a^	29.555 ± 2.86 ^bc^
	20	1.050 ± 0.18 ^a^	4.104 ± 1.16 ^a^	47.080 ± 3.09 ^a^

## Data Availability

The original contributions presented in the study are included in the article/Supplementary Materials, further inquiries can be directed to the corresponding author.

## Supplementary Materials

The following supporting information can be downloaded at: https://www.mdpi.com/article/10.3390/foods13152469/s1, Table S1: *cis*-regulatory elements of *AsMT1-4* promoters; Figure S1: comparison of the growth of *E. coli* cells transformed with empty pET21a(+) vector and pET21a(+) vectors harbouring coding regions of *AsMT1-4* in the presence of Zn and Cd ions. The relative growth rate is expressed as a slope of bacterial growth curves obtained by plotting optical density against time. Media were supplemented with two concentrations of Zn and Cd ions without IPTG. The results obtained for a given condition were compared, and distinct letters indicate significant differences between *E. coli* carrying different plasmids (Kruskal–Wallis, Mann–Whitney; *p* < 0.05).; Figure S2: photographs of oat plants inoculated and not inoculated with *Trichoderma viride* T5 growing in soil containing Cd after 6 months of growth.; Figure S3: Pearson correlation between shoot and root length, fresh and dry biomass, number of leaves, panicles and seeds, levels of Cu, Zn, and Cd, the amount of cadmium added to the soil, and the inoculation of oat seeds with *Trichoderma viride* T5 spores. Only significant correlations are shown. Figure S4: Pearson correlation between *A*s*MT1-4* expression (MT1-4), *Trichoderma viride* T5 inoculation, and the level of Cd in soil. Only significant correlations are shown.
