# Supplementary material for: The Contribution of Trichoderma viride and Metallothioneins in Enhancing the Seed Quality of Avena sativa L. in Cd-Contaminated Soil
Source: Foods. 2024 Aug 5;13(15):2469. doi: 10.3390/foods13152469 (PMC11312241; doi:10.3390/foods13152469)
Supplement: Supplementary file 1 [file foods-13-02469-s001.zip › Supplementary figures.pdf]

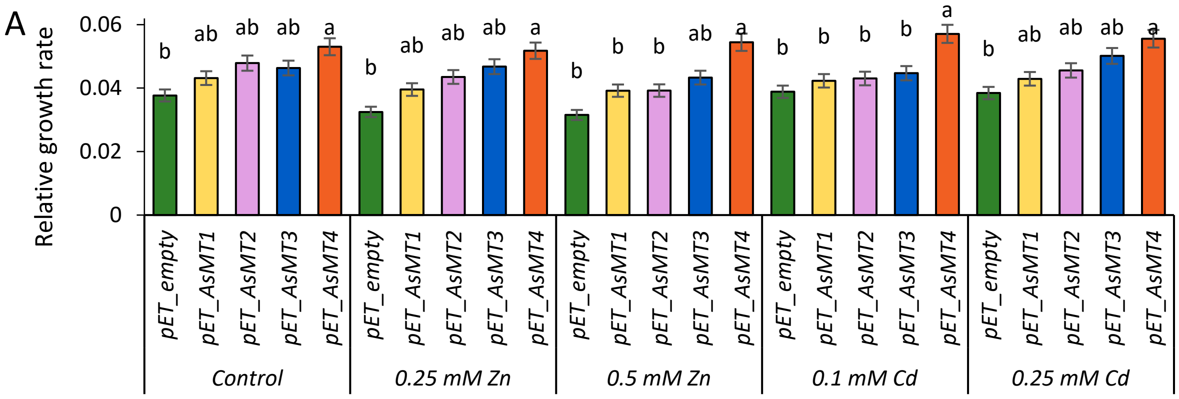

Supplementary Figure S1. Comparison of the growth of *E. coli* cells transformed with empty pET21a(+) vector and pET21a(+) vectors harbouring coding regions of AsMT1-4 in the presence of Zn and Cd ions. The relative growth rate is expressed as a slope of bacterial growth curves obtained by plotting optical density against time. Media were supplemented with two different concentrations of Zn and Cd ions without the addition of IPTG. The results obtained for a given condition were compared, and distinct letters indicate significant differences between *E. coli* carrying different plasmids (Kruskal– Wallis, Mann– Whitney; p <0.05).

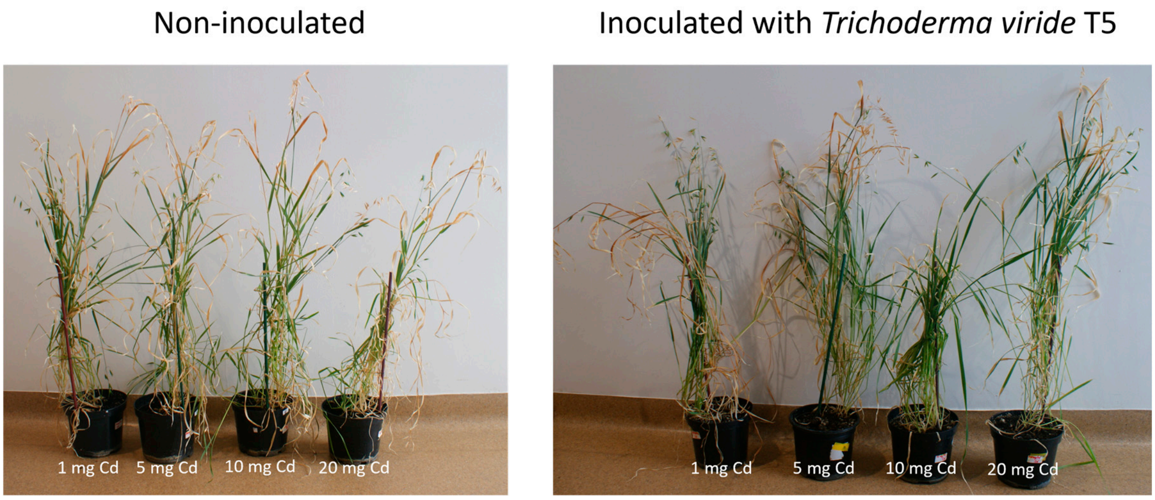

Supplementary Figure S2. Photographs of oat plants inoculated and non-inoculated with *Trichoderma viride* T5 growing in soil containing Cd after 6 moths of growth.

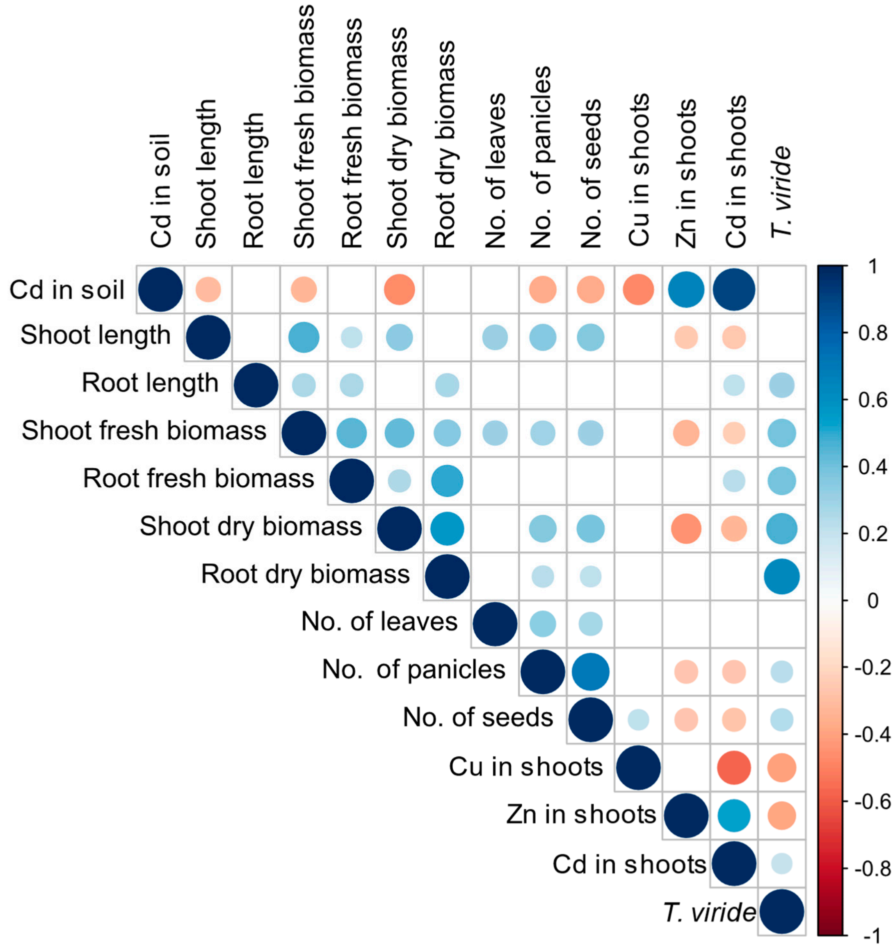

Supplementary Figure S3. Pearson correlation between shoot and root length, fresh and dry biomass, number of leaves, panicles and seeds, levels of Cu, Zn and Cd, the amount of cadmium added to the soil, and the inoculation of oat seeds with *Trichoderma viride* T5 spores. Only significant correlations are shown.

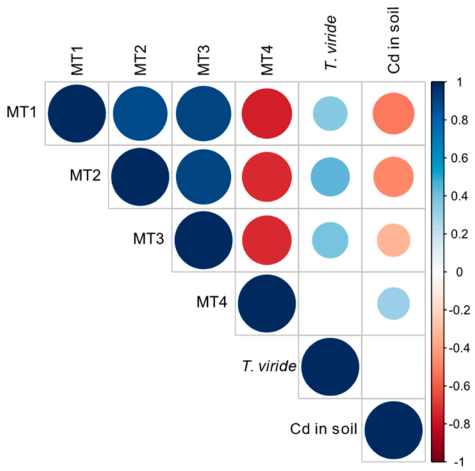

Supplementary Figure S4. Pearson correlation between *AsMT1-4* expression (MT1-4), *Trichoderma viride* T5 inoculation, and the level of Cd in soil. Only significant correlations are shown.
